# Non‐Hodgkin lymphoma presenting as acute pancreatitis: A rare occurrence

**DOI:** 10.1002/ccr3.1885

**Published:** 2018-11-20

**Authors:** Mitchell M. Pitlick, Jithma P. Abeykoon, Linda N. Dao, Carrie A. Thompson

**Affiliations:** ^1^ Department of Internal Medicine Mayo Clinic Rochester Minnesota; ^2^ Division of Hematopathology Mayo Clinic Rochester Minnesota; ^3^ Division of Hematology Mayo Clinic Rochester Minnesota

**Keywords:** CD20, extranodal manifestation, non‐Hodgkin lymphoma, pancreatitis

## Abstract

Lymphoma often presents with extranodal manifestations. However, pancreatic involvement resulting in pancreatitis is rare. CD20‐negative variants of diffuse large B‐cell lymphoma are also rare and are more likely to present with extranodal involvement. Lymphoma should be considered in patients presenting with pancreatitis without traditional risk factors.

## 1 CASE PRESENTATION

A 34‐year‐old woman was admitted to an outside hospital with severe epigastric abdominal pain. She had no report of cholelithiasis, history of alcohol use, elevated triglycerides, or use of drugs. She was diagnosed with acute pancreatitis on the basis of typical pain with a lipase of 1628 U/L (8‐78 U/L). She was treated conservatively and subsequently discharged. Following discharge, her pain never completely resolved. Therefore, MRI of the abdomen and pelvis was performed as an outpatient, which revealed mild heterogeneity and prominence of the pancreatic head with a trace amount of peri‐pancreatic fluid. She was readmitted to the hospital two weeks following the initial discharge due to worsening pain. Laboratories at this admission were significant for the following: AST, 597 U/L (8‐43 U/L); ALT, 1013 U/L (7‐45 U/L); total bilirubin, 5.2 mg/dL (<1.2 mg/dL); alkaline phosphatase, 695 U/L (50‐130 U/L); lipase 164 U/L (26‐102 U/L). She underwent endoscopic retrograde cholangiopancreatography, which showed a distal common bile duct stricture that was stented. CT of the abdomen and pelvis revealed multiple hypodense lesions in the liver, kidneys, pancreas, and anterior pericardium. She was subsequently transferred to our facility for further evaluation.

**Figure 1:**
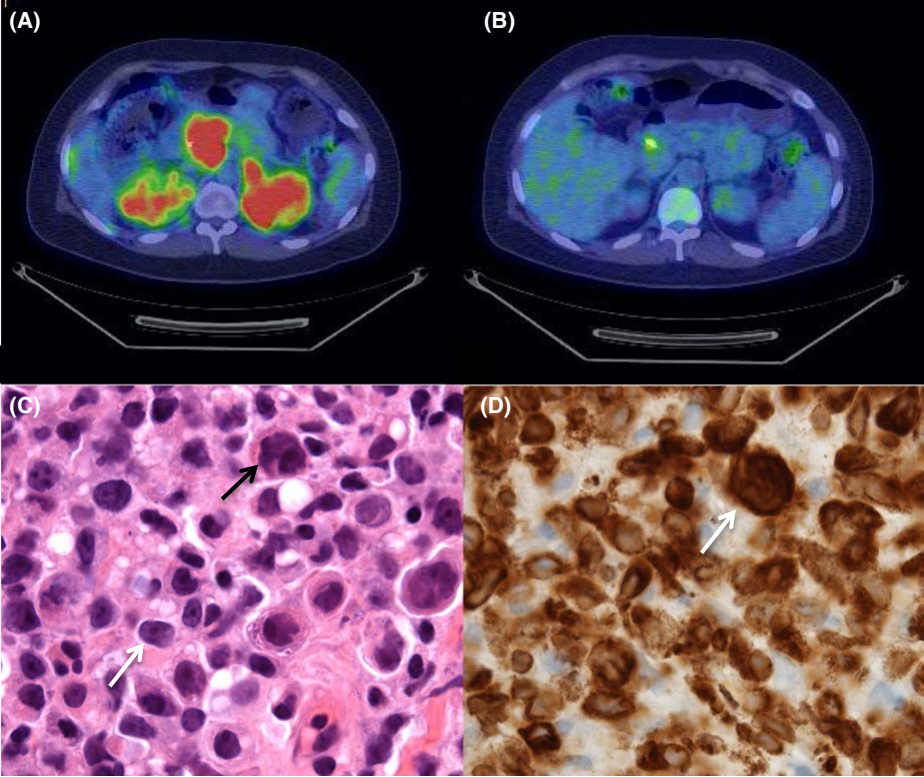
F18‐FDG PET and renal biopsy findings consistent with diffuse large B‐cell lymphoma. A, Widespread abdominal organ involvement prior to therapy. B, Significant interval improvement in disease burden after two cycles of CHOP. C, Kidney mass biopsy showing lymphoid cells with large nuclei, irregular nuclear contours, prominent nucleoli, and modest amounts of cytoplasm (white arrow) with scattered forms containing very large, pleomorphic nuclei (black arrow) (hematoxylin and eosin stain, 100x). D, Tumor cells positive for CD79a (white arrow) (CD79a immunoperoxidase stain, 100x)

At the time of transfer, the patient complained of severe epigastric and right upper quadrant pain as well as intense generalized pruritus. She also complained of drenching sweats and a 12‐pound weight loss. Additional laboratory testing revealed an LDH of 486 U/L (122‐222 U/L). Ultrasound‐guided biopsy of a renal mass showed an abnormal lymphoid infiltrate with abundant necrosis. The infiltrate contained lymphoid cells with large nuclei, irregular nuclear contours, prominent nucleoli, and modest amounts of cytoplasm. There were scattered forms with very large, pleomorphic nuclei (hematoxylin and eosin stain, Figure 1C). The tumor cells were positive for CD79a, PAX5, CD19, CD22, OCT2, BCL‐6, MYC, CD30, and CD45 (CD79a immunoperoxidase stain, Figure 1D). They were negative for CD20, MUM‐1, CD10, and BCL‐2. FISH analysis did not show a MYC rearrangement. A final diagnosis of CD20‐negative diffuse large B‐cell lymphoma was made. She was discharged following adequate control of her pain and pruritus.

Further staging was performed as an outpatient, including F18‐FDG PET scan, which showed intense FDG uptake in the anterior mediastinal mass, bilateral renal masses, pancreas, supraclavicular lymph nodes, middle mediastinal lymph nodes, bilateral adrenal glands, anterior left iliac bone, and the T6 vertebral body (Figure 1A). Analysis of bone marrow and cerebrospinal fluid was negative for lymphoma involvement. She was deemed to have Ann Arbor stage IVB disease with an International Prognostic Index of 3. As such, she was initiated on CHOP [cyclophosphamide (750 mg/m^2^, intravenous), doxorubicin (50 mg/m^2^, intravenous), vincristine (1.4 mg/m^2^, intravenous), and prednisone (100 mg/m^2^, oral)] with methotrexate (3.5 gm/m^2^, intravenous) included during odd cycles for CNS prophylaxis. A repeat F18‐FDG PET scan after three cycles of CHOP showed marked interval improvement with reduction in size and FDG avidity of all previously demonstrated masses (Figure 1B).

## 2 DISCUSSION

Non‐Hodgkin lymphoma frequently involves extra‐nodal sites, although pancreatic involvement is quite rare, being found in only 0.2%‐2% of patients at presentation.^1^, ^2^ In addition, secondary pancreatic involvement by non‐Hodgkin lymphoma presenting as acute pancreatitis is an even more rare occurrence, with very few reported cases in the literature.^2^, ^3^ Primary pancreatic lymphoma presenting as acute pancreatitis is also seldom encountered, with cases occurring in the setting of discrete masses^4^ as well as with diffuse infiltrative processes.^5^ While the majority of diffuse large B‐cell lymphomas are CD20‐positive, 1%‐2% are actually CD20‐negative.^6^, ^7^ CD20‐negative variants tend to be more aggressive and more often present with extranodal involvement compared to their CD20 counterparts.^6^, ^7^ Lymphoma is often not thought of in the differential diagnosis of pancreatitis. It should be considered in the differential diagnosis in patients presenting with acute pancreatitis with no obvious cause and in whom symptoms persist or worsen despite adequate therapy.

## CONFLICT OF INTEREST

Authors have nothing to disclose.

## AUTHOR CONTRIBUTION

MMP and JPA: prepared the manuscript and critically reviewed the manuscript. LND: involved in the pathology interpretation, preparation of figures, and critical review of the manuscript. CAT: critically reviewed the manuscript.
